# The Factors Influencing the Efficacy of Pregabalin Combined With Lidocaine in the Treatment of Postherpetic Neuralgia Patients: An Analysis

**DOI:** 10.1002/iid3.70339

**Published:** 2026-02-12

**Authors:** Xinkai Wang, Weiliang Cong, Jian Jin, Lin Zhao

**Affiliations:** ^1^ Department of Pain The Third Affiliated Hospital of Qiqihar Medical University Qiqihar China; ^2^ Department of Pain The First Affiliated Hospital of China Medical University Shenyang China

**Keywords:** Herpes Zoster, influencing factors, lidocaine, neuralgia, pregabalin

## Abstract

**Objective:**

This study aims to analyze the primary factors influencing the therapeutic effect of pregabalin combined with lidocaine in patients with post‐herpetic neuralgia (PHN).

**Methods:**

Clinical data of 97 PHN patients admitted to our hospital from January 2022 to October 2024 were retrospectively analyzed. All patients received pregabalin combined with lidocaine treatment. Treatment efficacy was assessed using the numerical rating scale (NRS) for pain, dividing patients into effective (≥ 50% NRS reduction) and ineffective groups. Univariate and multivariate logistic regression analyses identified the main influencing factors. Pearson correlation analysis explored relationships between factors and therapeutic effect. Receiver operating characteristic (ROC) curves evaluated predictive values.

**Results:**

Posttreatment, 77 patients (79.38%) were classified as responders, while 20 (20.62%) were nonresponders. Age (OR: 0.163, 95% CI: 0.036–0.535), illness duration (OR: 0.098, 95% CI: 0.015–0.369), pain severity (OR: 2.794, 95% CI: 1.362–6.566), and presence of concomitant immunosuppressive diseases (OR: 0.194, 95% CI: 0.030–0.742) were key determinants. Positive correlations were found between illness duration (*r* = 0.352), pain severity (*r* = −0.279), immunosuppressive diseases (*r* = 0.231), and treatment effectiveness (*p* < 0.05). Age showed a negative correlation (*r* = 0.301, *p* < 0.05). ROC curves indicated AUCs for age, illness duration, pain severity, and immunosuppressive diseases were 0.685, 0.716, 0.691, and 0.368, respectively. Combined, these factors had a higher predictive value (AUC = 0.734).

**Conclusion:**

Age ≥ 65 years, illness duration ≥ 3 months, severe pain, and the presence of concomitant diseases causing immunosuppression are the main factors influencing the effectiveness of combined pregabalin and lidocaine treatment for PHN.

## 1 Introduction

Herpes zoster is a disease caused by the reactivation of varicella‐zoster virus (VZV) [1]. This reactivation results in neural inflammation, necrosis, and the development of post‐herpetic neuralgia (PHN), which significantly impairs patients' quality of life and functional capacity [1, 2]. PHN is characterized by persistent neuropathic pain lasting beyond the healing of the acute herpetic rash, often presenting a substantial therapeutic challenge due to its complex pathophysiology and variable response to existing treatments [3, 4].

Currently, pharmacological intervention is a widely adopted strategy for managing PHN, though treatment responses vary across different agents. Pregabalin, a calcium channel modulator, and lidocaine, a local anesthetic, are commonly used either alone or in combination for neuropathic pain control [5, 6]. Previous studies have suggested that the combination of pregabalin and lidocaine may exert synergistic effects in alleviating PHN [7]. Nonetheless, a subset of patients exhibits suboptimal responses to this regimen, highlighting the need to investigate factors that may influence treatment outcomes. Identifying such factors could aid in personalizing therapy and improving overall efficacy. Therefore, this study analyzed the influencing factors of the therapeutic effectiveness of combined pregabalin and lidocaine treatment in PHN patients, aiming to provide effective references for the clinical management of PHN patients. By examining clinical data and patient characteristics, we sought a comprehensive understanding to elucidate decisive factors for the efficacy of this combination treatment method.

## 2 Methods and Materials

### 2.1 Study Population

A retrospective analysis was conducted on the clinical data of 97 patients with PHN admitted to our hospital from January 2022 to October 2024. Inclusion criteria were as follows: (1) meeting the diagnostic criteria for PHN outlined in the “Clinical Preface Model Guidelines for Herpes Zoster‐Related Pain and Postherpetic Neuralgia [8],” which includes a clear history of herpes zoster, persistent pain lasting for over 4 weeks after the disappearance of the herpes zoster rash; (2) having a relatively stable mental state and being able to actively participate in the study. Exclusion criteria were: (1) use of corticosteroids or immunosuppressive drugs in the month preceding the study; (2) presence of multi‐organ dysfunction such as liver, kidney, or heart disorders; (3) concomitant hematological disorders; (4) presence of other severe diseases or conditions that could affect laboratory tests; (5) poor tolerance to the treatment drugs used in this study; and (6) loss of research data. This study was conducted in accordance with the principles of the Helsinki Declaration and approved by the Medical Ethics Committee of Third Affiliated Hospital of Qiqihar Medical University (Approval No. 2025LL‐86).

### 2.2 Treatment Regimen

All patients received combined treatment of pregabalin and lidocaine, administered as follows: using 5% lidocaine gel patch (Beijing Tide Pharmaceutical Co. Ltd., National Drug Approval H20180007) in combination with pregabalin capsules (Pfizer Pharmaceuticals Limited, National Drug Approval J20100102). The usage of 5% lidocaine gel patch was as follows: cut the patch into pieces and apply it to the most intense pain area (up to 3 patches at a time), with a maximum cumulative application time of 12 h within 24 h, for a total of 4 weeks. The usage of pregabalin capsules was as follows: 300 mg daily, administered orally twice daily, for a treatment duration of 4 weeks.

### 2.3 Assessment of Therapeutic Effect

The effectiveness of the treatment was evaluated using the numerical rating scale (NRS) for pain after 4 weeks. Patients were instructed to self‐rate their pain on a 10‐point scale based on the intensity of pain, where 0 represents no pain, 1–3 indicates mild pain, 4–6 indicates moderate pain, 7–9 indicates severe pain, and 10 indicates unbearable and intense pain [9]. Pain relief was defined as *a* ≥ 50% reduction in NRS score at 3 months after treatment compared to before treatment, and patients were categorized accordingly, with those experiencing pain relief placed in the effective group and those without pain relief in the ineffective group. The effectiveness rate was calculated as the number of effective cases divided by the total number of cases, multiplied by 100%, while the ineffectiveness rate was calculated as the number of ineffective cases divided by the total number of cases, multiplied by 100%.

### 2.4 Data Collection

Patient data, including gender, age, affected area, duration of illness, severity of pain, presence of concomitant diseases causing immunosuppression (malignant tumors, fractures, renal failure, respiratory tract infections, rheumatism, cerebral infarction, traumatic brain injury, and postthoracotomy), pain hypersensitivity (assessed by gently brushing the local skin lesion area with a cotton swab at a speed of 1–2 cm/s, with pain elicited if present), and occurrence of paroxysmal pain, were retrospectively collected from medical records during the patients' experience of pain [10].

### 2.5 Statistical Methods

Data analysis was performed using SPSS 25.0 statistical software, representing categorical data as numbers and utilizing the *χ* ^2^ test. The independent variables included gender, age, affected area, duration of illness, severity of pain, presence of concomitant diseases causing immunosuppression, pain hypersensitivity, and occurrence of paroxysmal pain. The Spearman rank correlation was used to explore the relationship between factors and therapeutic effectiveness for those exhibiting differences. Subsequently, binary logistic regression analysis was conducted, with *p* < 0.05 indicating statistical significance. The NRS score was used as the test variable, with treatment outcome as the state variable. Receiver operating characteristic (ROC) curves were generated, and the sensitivity, specificity, area under the curve (AUC), and Youden index were calculated to analyze the predictive value of each factor for the therapeutic effectiveness of pregabalin combined with lidocaine treatment.

## 3 Results

### 3.1 Factors Affecting the Therapeutic Effect of Pregabalin Combined With Lidocaine Treatment

As shown in Table 1, out of the 97 patients, 77 demonstrated effectiveness after treatment and were included in the effective group, accounting for 79.38% (77/97); the remaining 20 patients were classified as ineffective, making up 20.62% (10/97). There were no statistically significant differences between the two groups in terms of gender, affected area, pretreatment pain hypersensitivity, prior antiviral treatment, and paroxysmal pain (*p* > 0.05). However, there were statistically significant differences in terms of age, duration of illness, severity of pain, and presence of concomitant diseases causing immunosuppression (*p* < 0.05), indicating that age, duration of illness, severity of pain, and the presence of concomitant diseases causing immunosuppression may be influencing factors for the therapeutic effect of pregabalin combined with lidocaine treatment in patients with PHN.

**Table 1:** Baseline characteristics and univariate analysis of PHN patients treated with pregabalin combined with lidocaine.

Factor	Cases	Effective group (*n* = 77)	Ineffective group (*n* = 20)	*χ* ^*2*^	*p* value
Gender					
Male	58	45	13	0.077	0.782
Female	39	32	7		
Age					
< 65 years	43	40	3	7.349	0.007
≥ 65 years	54	37	17		
Lesion site					
Axilla	9	7	2		0.979
Back	28	22	6		
Chest	26	20	6		
Head and face	15	13	2		
Lumbosacral region	19	15	4		
Illness duration					
< 3 months	43	41	2	10.343	0.001
≥ 3 months	54	36	18		
Severity of pain					
Mild (VAS < 4)	24	22	2	8.724	0.013
Moderate (4 ≤ VAS < 7)	33	29	4		
Severe(VAS ≥ 7)	40	26	14		
Pretreatment pain hypersensitivity					
None	60	47	13	0.004	0.947
Present	37	30	7		
Prior antiviral treatment					
None	55	43	12	0.007	0.935
Present	42	34	8		
Presence of concomitant diseases Causing immunodeficiency					
None	30	28	2	4.005	0.045
Present	67	49	18		
Paroxysmal pain					
≤ 3 times	46	38	8	0.245	0.621
> 3 times	51	39	12		

*Note:* Data are presented as the number of patients. Categorical variables were compared using the *χ*² test.

Abbreviations: PHN, post‐herpetic neuralgia; VAS, visual analog scale.

*p* < 0.05 indicates statistical significance.

### 3.2 Multifactorial Analysis of the Effects of Pregabalin Combined With Lidocaine Treatment in PHN Patients

In the multifactorial analysis, age, duration of illness, severity of pain, and the presence of immunocompromised diseases were designated as independent variables X1, X2, X3, and X4, with values assigned as follows: X1 ≥ 65 years, X2 ≥ 3 months, X3 severe pain, and X4 the presence of concomitant diseases causing immunosuppression were assigned a value of 1, while the remaining were assigned a value of 0. As shown in Table 2, according to the logistic regression equation, age (OR: 0.163, 95% CI: 0.036–0.535), duration of illness (OR: 0.098, 95% CI: 0.015–0.369), severity of pain (OR: 2.794, 95% CI: 1.362–6.566), and the presence of concomitant diseases causing immunosuppression (OR: 0.194, 95% CI: 0.030–0.742) were identified as the main factors influencing the therapeutic effect of combined pregabalin and lidocaine treatment in PHN patients.

**Table 2:** Multifactorial analysis of the therapeutic effect of pregabalin combined with lidocaine treatment in PHN patients.

Factor	*p* value	OR	95% CI of OR
Age	0.007	0.163	0.036–0.535
Illness duration	0.003	0.098	0.015–0.369
Severity of pain	0.009	2.794	1.362–6.566
Presence of concomitant diseases causing immunodeficiency	0.036	0.194	0.030–0.742

*Note:* Binary logistic regression was performed with treatment effectiveness as the dependent variable.

Abbreviations: CI, confidence interval; OR, odds ratio; PHN, post‐herpetic Nneuralgia.

### 3.3 Correlation Analysis of Factors and Treatment Effect

**Figure 1:**
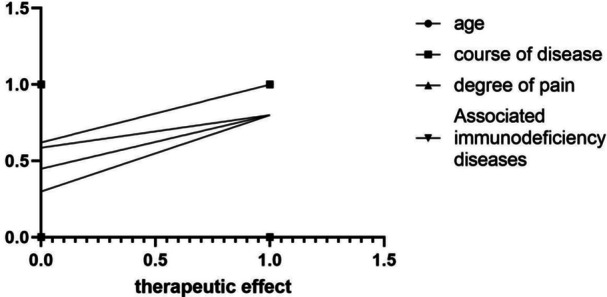
Heatmap of the correlation analysis between factors and treatment effectiveness.

As shown in Table 3 and Figure 1, Spearman's rank correlation analysis revealed that the duration of illness, severity of pain, the presence of concomitant diseases causing immunosuppression, and treatment effectiveness were all positively correlated (*r* = 0.352, −0.279, 0.231, *p* < 0.05). Conversely, age and the presence of concomitant diseases exhibited a negative correlation with treatment effectiveness (*r* = 0.301, *p* < 0.05). These findings demonstrate a significant correlation between age, duration of illness, severity of pain, the presence of concomitant diseases causing immunosuppression, and treatment effectiveness.

**Table 3:** Correlation analysis of factors with treatment effectiveness.

Factor	*r*	*p* value
Age	0.301	0.003
Illness duration	0.352	*p* < 0.001
Severity of pain	0.279	0.006
Presence of concomitant diseases causing immunodeficiency	0.231	0.023

*Note:* Spearman's rank correlation analysis was used. *r*, correlation coefficient; *p* < 0.05 indicates statistical significance.

### 3.4 Predictive Value of Individual or Combined Factors for the Therapeutic Effect of Pregabalin Combined With Lidocaine Treatment in PHN Patients

**Figure 2:**
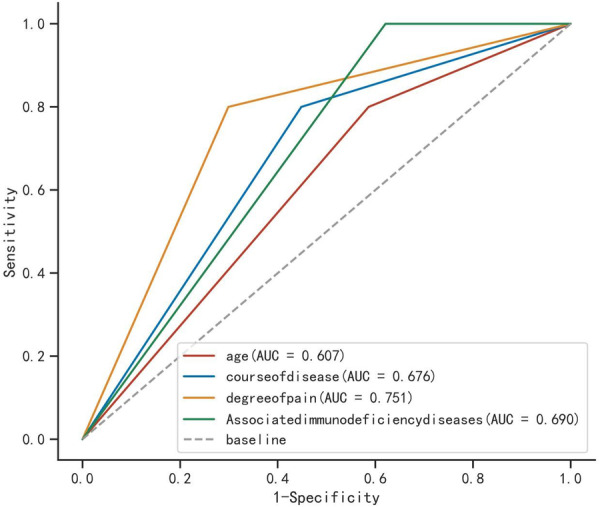
ROC curves of individual or combined factors predicting the therapeutic effect of pregabalin combined with lidocaine in PHN patients.

As depicted in Table 4 and Figure 2, the ROC curves revealed that the AUC for age, duration of illness, severity of pain, and presence of concomitant diseases causing immunosuppression were 0.685, 0.716, 0.691, and 0.368, respectively. When combined, the AUC for all factors was 0.734, indicating that the combined factors can enhance the predictive value for the therapeutic effect of pregabalin combined with lidocaine treatment in PHN patients.

**Table 4:** Predictive value of individual or combined factors for the therapeutic effect of pregabalin combined with lidocaine treatment in PHN patients.

Factor	AUC value	Sensitivity	Specificity	Youden index
Age	0.685	0.850	0.519	0.369
Illness duration	0.716	0.900	0.532	0.432
Severity of pain	0.691	0.700	0.662	0.362
Presence of concomitant diseases causing immunodeficiency	0.368	1	0	0
Combined	0.734	1.000	0.468	0.468

*Note:* Receiver operating characteristic (ROC) curves were generated for each factor and their combination.

Abbreviation: AUC, area under the curve; Youden index = sensitivity + specificity − 1.

## 4 Discussion

The management of PHN continues to pose a considerable therapeutic challenge, necessitating treatment strategies that are both effective and well‐tolerated [11]. The combination of systemic pregabalin and topical lidocaine has gained traction based on its multimodal mechanism of action, targeting both central sensitization (via pregabalin) and peripheral nociceptor hyperactivity (via lidocaine) [12, 13]. While this approach is rational and supported by clinical guidelines, a uniform treatment response is not observed, highlighting a gap between theoretical efficacy and real‐world effectiveness that is likely mediated by heterogeneous patient characteristics [14]. Our study focused on elucidating these modulating factors within a cohort of 97 PHN patients treated with this combination.

Our results demonstrated an overall effectiveness rate of 79.38%, which aligns with the spectrum of efficacy rates reported in comparable studies but sits at the mid‐to‐lower end of the range [15, 16]. For instance, some trials report success rates above 85%, while others note markedly nonresponse [17]. This variability across studies can be attributed to differences in population demographics, diagnostic criteria, treatment protocols (including dosing and duration), and, critically, the definitions used for “treatment success.” The 20.62% nonresponse rate in our cohort underscores the clinical reality that a meaningful subset of patients does not achieve adequate relief, reinforcing the imperative to identify predictors of poor outcome.

Multivariate logistic regression analysis identified four independent clinical factors markedly associated with reduced likelihood of treatment success: advanced age (≥ 65 years), longer illness duration (≥ 3 months), higher baseline pain severity, and the presence of concomitant conditions associated with immunosuppression. The odds ratios for age and illness duration were particularly notable, indicating a strong negative influence on therapeutic outcome.

The negative correlation between advanced age and treatment efficacy is physiologically plausible. Aging is associated with a natural decline in neuronal regenerative capacity, reduced reserve in both cellular and humoral immune responses to viral reactivation, and a higher prevalence of comorbidities such as diabetes or vascular disease that can impair nerve healing [18, 19]. These factors collectively may create a physiological milieu less conducive to pain resolution with standard pharmacological interventions. This finding corroborates several epidemiological studies linking older age with more severe and persistent PHN [20]. However, it contrasts with some clinical trials where age was not a marked predictor, possibly due to stricter inclusion criteria or younger overall sample populations [21]. Future research should employ age‐stratified analyses to clarify its role across different life decades.

Similarly, a longer duration of PHN before initiation of the combination therapy was a robust predictor of poorer response. This supports the well‐established concept in pain medicine of “pain chronicity.” Prolonged nociceptive input drives neuroplastic changes, including central sensitization in the spinal cord and brain, wind‐up phenomena, and possible structural reorganization of synaptic connectivity [22]. Over time, the pain state may become less dependent on the initial peripheral trigger and more sustained by self‐perpetuating central mechanisms, which may be less responsive to drugs primarily targeting peripheral or early synaptic events. While some studies have not found illness duration to be notable, this may be because their cohorts comprised patients with a relatively narrow and shorter duration range [23]. Our findings strongly suggest that early intervention, before the establishment of entrenched central sensitization, is likely critical for maximizing therapeutic success.

The severity of baseline pain also emerged as a marked independent factor. Patients reporting severe pain (NRS ≥ 7) were less likely to achieve a half pain reduction. This may indicate that higher pain intensity reflects a greater magnitude of neuropathological burden—more extensive nerve damage, stronger inflammatory cascades, or more profound central sensitization—which may overwhelm the modulatory capacity of the drug combination [24]. Alternatively, severe pain might represent a distinct phenotypic subtype of PHN with a different underlying mechanism. Although some previous work has not identified pain intensity as a predictor, methodological differences in pain assessment or a ceiling effect in milder cohorts could explain the discrepancy [25].

Furthermore, the presence of concomitant conditions that induce a state of immunosuppression (e.g., active malignancy, chronic renal failure, or long‐term corticosteroid use) was associated with worse outcomes. Immune competence plays a dual role in PHN: it is crucial for controlling viral replication initially and is increasingly recognized as a modulator of neuroinflammation and nerve repair thereafter [26]. An immunosuppressed state may allow for more extensive viral damage during the acute phase and impair subsequent healing and resolution of inflammation, leading to a more severe and treatment‐resistant neuropathic pain state. This factor, often overlooked in purely neurological assessments, underscores the importance of a holistic patient evaluation.

Importantly, while each factor alone had modest to moderate predictive value, their combination yielded a higher AUC. This demonstrates that treatment response is multifactorial, and a composite clinical profile, integrating age, chronicity, pain severity, and immune status, provides a more accurate prognostic picture than any single variable [27]. This aligns with the modern shift towards multidimensional assessment in chronic pain management.

Several limitations of this study warrant consideration. Its retrospective, single‐center design may introduce selection bias and limit the generalizability of the findings. The sample size, while adequate for the statistical analyses performed, is modest, and validation in a larger, multicenter prospective cohort is essential. Furthermore, our analysis was confined to readily available clinical variables; future investigations would benefit from incorporating quantitative sensory testing, biomarkers of inflammation or neural damage, and genetic polymorphisms to build a more comprehensive predictive model. The definition of “immunosuppression” was based on clinical diagnoses, and a more granular assessment of immune function was not available.

## 5 Conclusion

In conclusion, age ≥ 65 years, illness duration ≥ 3 months, severe baseline pain, and comorbid immunosuppressive conditions were identified as key factors influencing the effectiveness of pregabalin combined with lidocaine in PHN patients. Clinical practitioners should consider these factors when selecting and monitoring therapy. Future research should focus on larger, prospective cohorts, integrate multimodal biomarkers, and develop individualized prediction models to optimize therapeutic outcomes in PHN.

## Author Contributions

Xinkai Wang designed the manuscript. Xinkai Wang and Weiliang Cong provided the administrative support. Xinkai Wang and Lin Zhao provided the materials. Weiliang Cong and Jian Jin collected the data. Xinkai Wang analyzed the data. All authors wrote the manuscript. All authors reviewed the manuscript.

## Ethics Statement

This study was conducted in accordance with the principles of the Helsinki Declaration and approved by the Medical Ethics Committee of Third Affiliated Hospital of Qiqihar Medical University (Approval No. 2025LL‐86).

## Conflicts of Interest

The authors declare no conflicts of interest.

## Data Availability

The data that support the findings of this study are available from the corresponding author upon reasonable request.
